# Supplementary figures and images for: STRIPAK complex defects result in pseudosexual reproduction in Cryptococcus neoformans
Source: PLoS Genet. 2025 Jun 30;21(6):e1011774. doi: 10.1371/journal.pgen.1011774 (PMC12240305; doi:10.1371/journal.pgen.1011774)

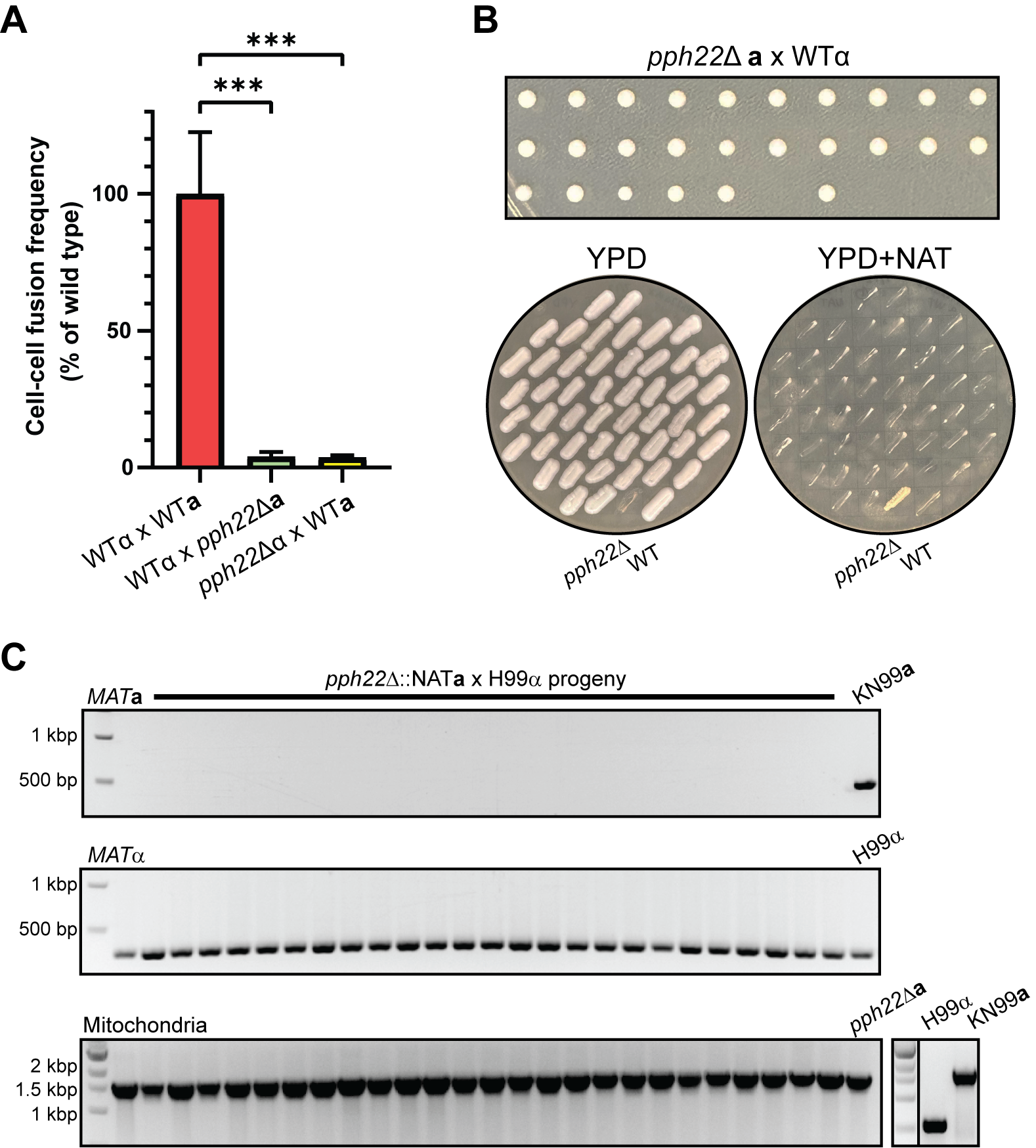

Supplement: S1 Fig — A) Cell-cell fusion assay of WT::NAT x WT::NEO and pph22Δ::NAT x WT::NEO crosses. Cells were cocultured on MS media for three days before harvesting and plating onto selective media. Frequencies are expressed as a percentage of the wild-type control cross. Results represent three independent experiments. Statistical significance was calculated using one-way ANOVA with Dunnett’s multiple comparisons test (***, P < 0.001). B) Dissection of progeny from pph22Δ x WT on YPD. Each row is from a separate basidium. Germinated progeny were transferred to YPD and YPD + NAT. The pph22Δ and wild-type parental strains were included as controls. pph22Δ exhibits almost no growth on YPD due to its inherent growth defects and being outcompeted for nutrients with the surrounding wild-type strains. C) Example gels from PCR genotyping showing pph22Δ x WT possess only one mating type and inherited mitochondria from the MATa parent. H99α, KN99a, and pph22Δ strains served as controls. (TIF) [file pgen.1011774.s001.tif]

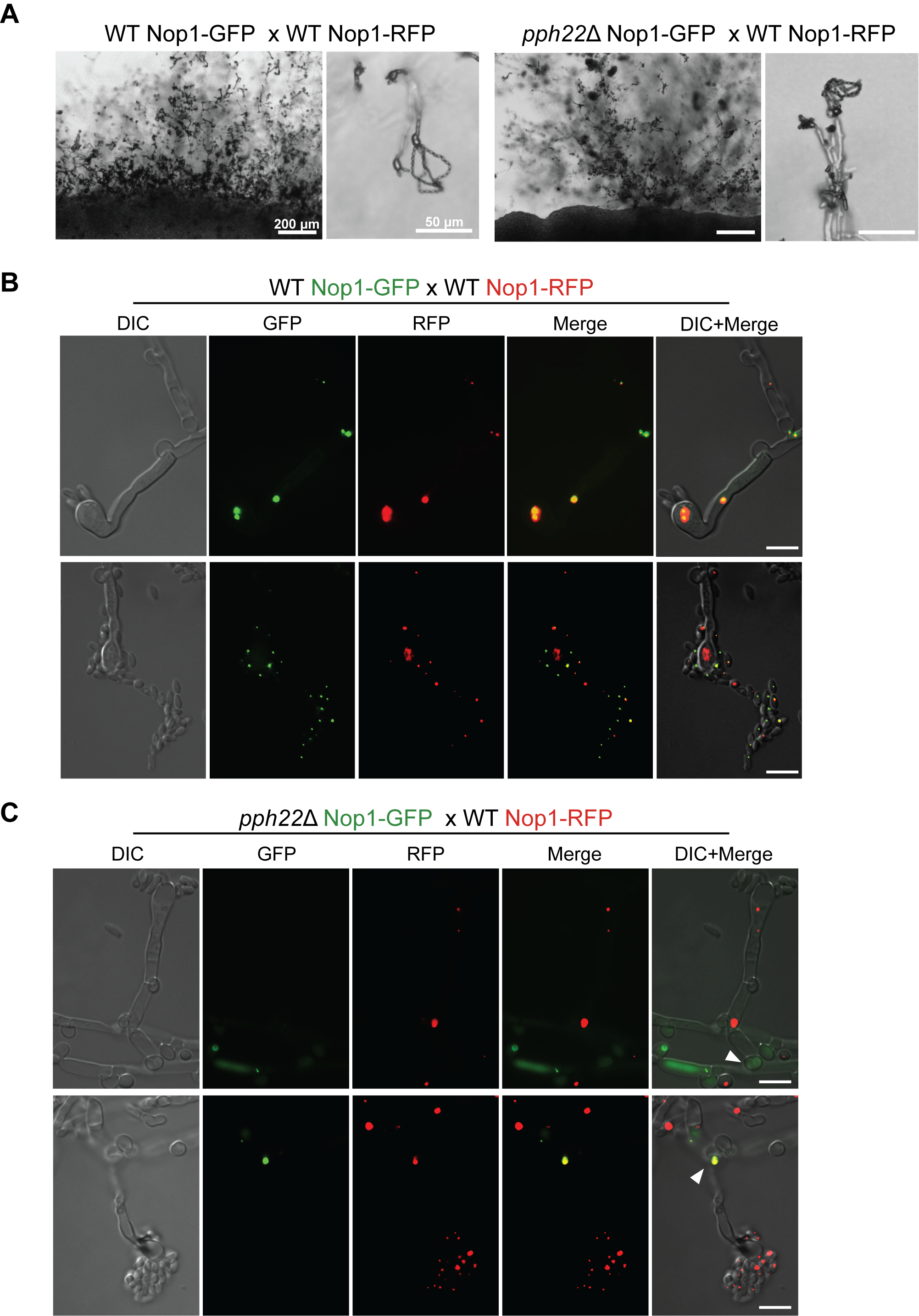

Supplement: S2 Fig — A) Mating of wild-type and pph22Δ strains expressing fluorescently-tagged NOP1 on MS media. A) WT (H99α) or pph22Δ expressing NOP1-GFP was crossed with WT (KN99a) expressing NOP1-mCherry (RFP) and mated on MS medium. B) The indicated strains expressing NOP1-GFP and NOP1-RFP were mated on MS plates for 6–8 weeks before imaging. DIC and fluorescence images were captured with live cells. The scale bar in each panel represents 5 μm. (TIF) [file pgen.1011774.s002.tif]

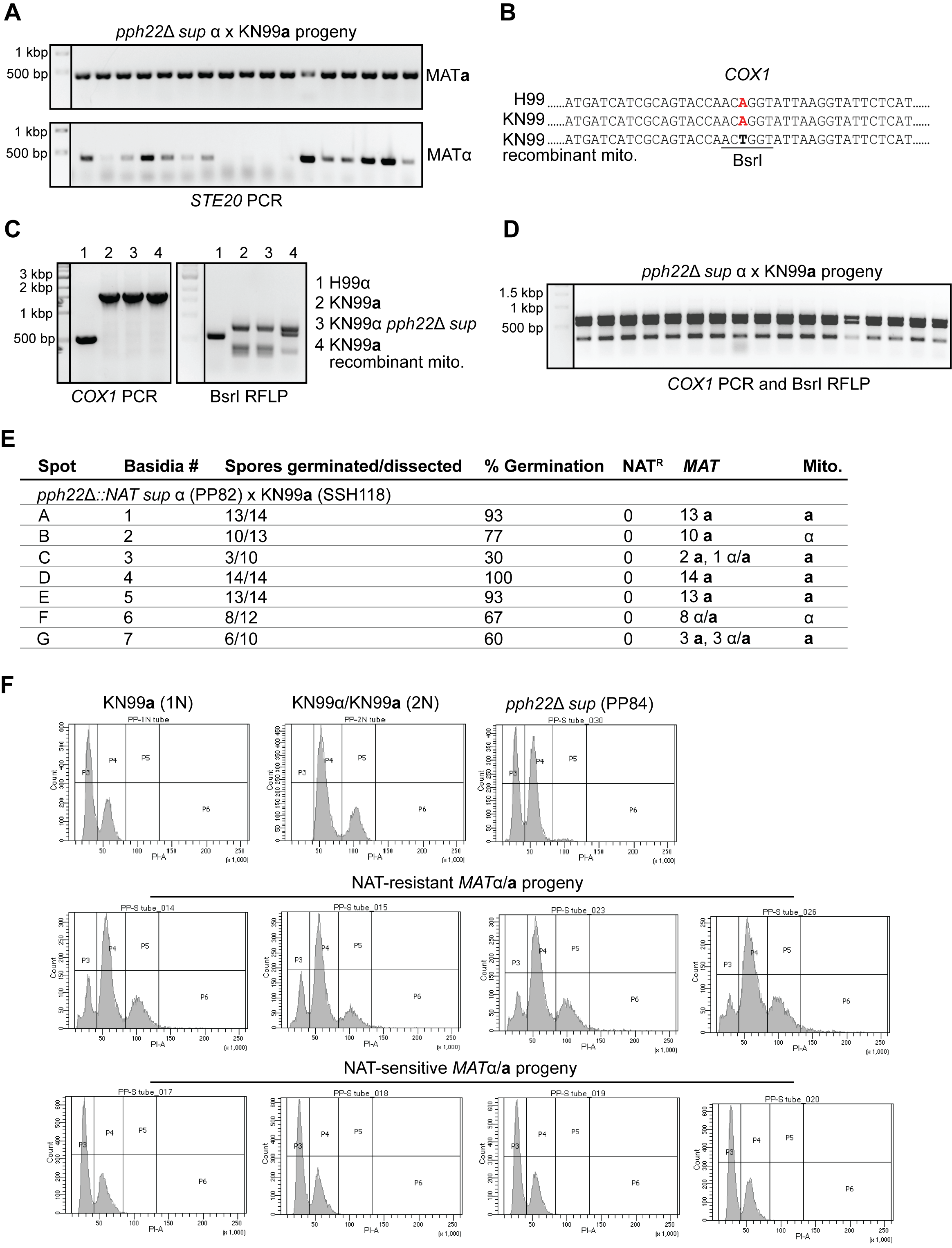

Supplement: S3 Fig — A) Representative agarose gel electrophoresis analysis of PCR amplification of the STE20 gene in pph22Δ sup x WT progeny shows heterozygosity of the MAT allele. B) Single nucleotide polymorphism in the COX1 gene in the KN99 strain with recombinant mitochondria generates a BsrI restriction enzyme recognition site. C) Example of COX1 fragment from PCR before and after BsrI digestion in the indicated strains used form RFLP analysis. D) RFLP following BsrI digestion of the COX1 PCR products from pph22Δ sup x WT progeny. E) Genotype analysis of progeny from a third independent suppressor strain of pph22Δ sup crossed with wild type. F) FACS analysis of heterozygous α/a progeny along with KN99a (1N), CnLC6683 (2N), and PP84 as controls. (TIF) [file pgen.1011774.s003.tif]

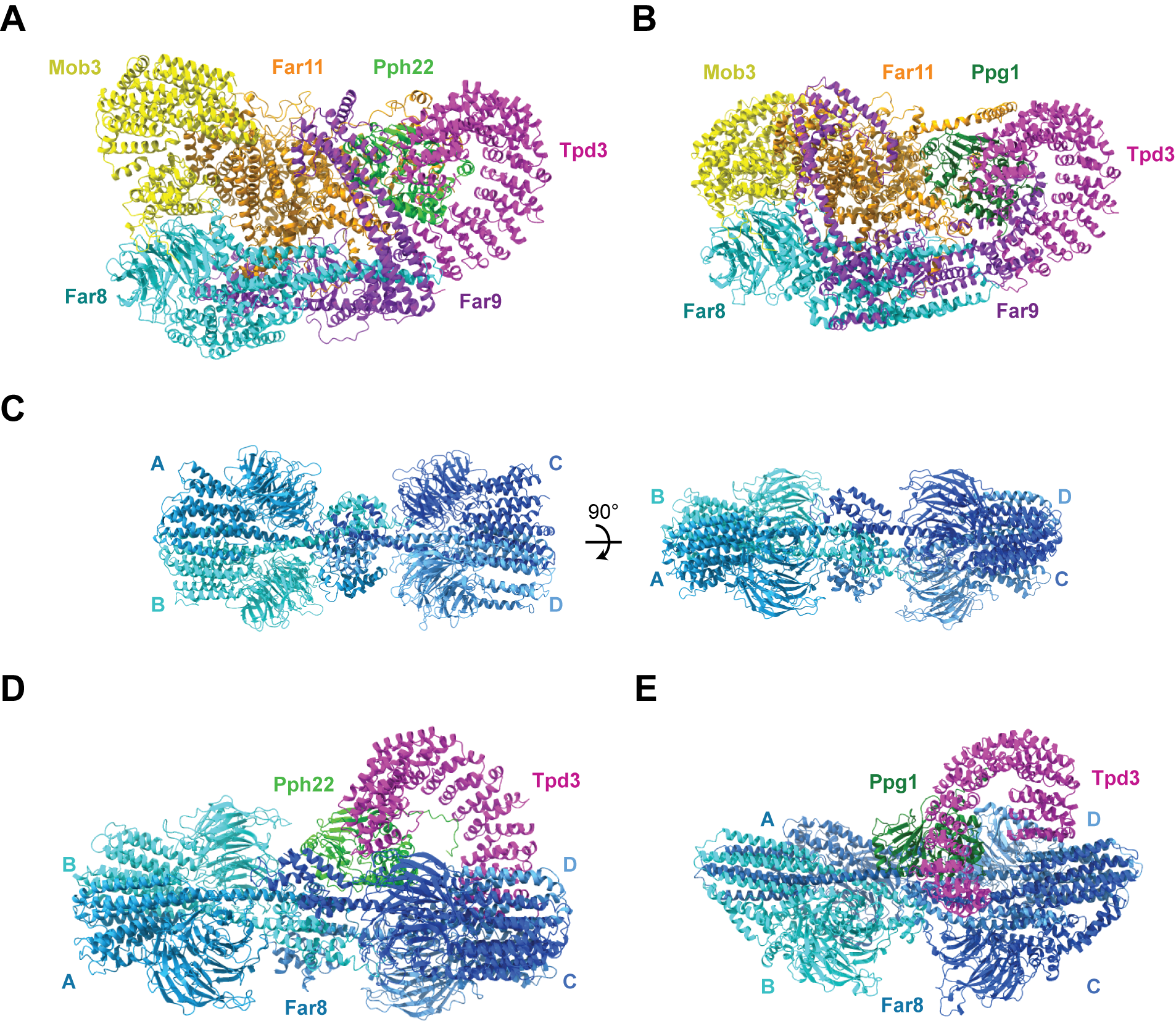

Supplement: S4 Fig — Models of STRIPAK with Pph22 (A) or Ppg1 (B) serving as the catalytic subunit. C) Homo-tetramer prediction of Far8, with protein monomers labeled A-D. The predicted multi-modular PP2A complex with Pph22 (D) or Ppg1 (E). (TIF) [file pgen.1011774.s004.tif]

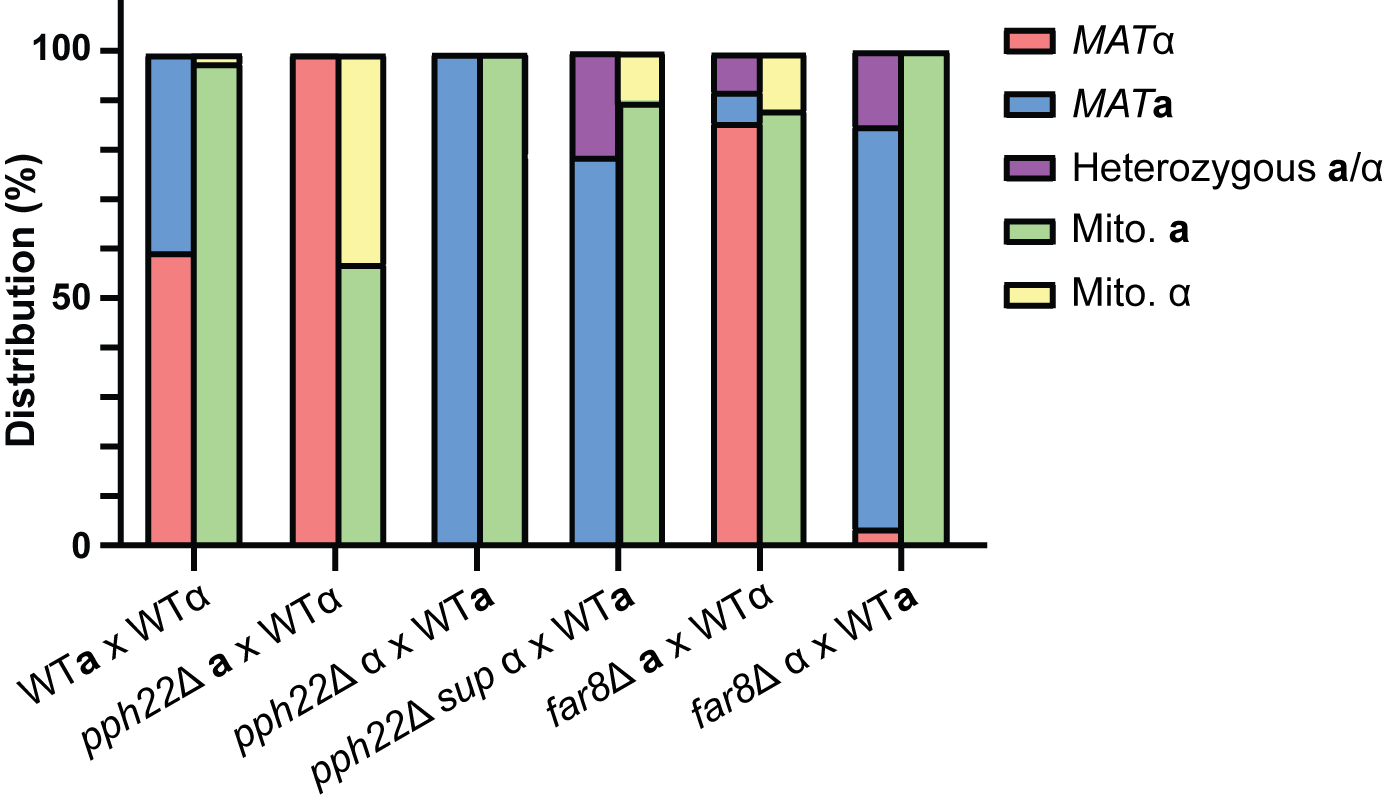

Supplement: S5 Fig — Distribution of genotypes among progeny in wild-type, pph22Δ, pph22Δ sup, and far8Δ crosses. Number of progeny analyzed for each cross are (from left to right): 58, 282, 112, 281,122, 55. Fisher’s exact test was performed for both mating type and mitochondrial type of pph22Δ/pph22Δ sup vs. WT and far8Δ vs. WT comparison groups, revealing a significant deviation in the distribution of genotypes among progeny from pph22Δ, pph22Δ sup, and far8Δ crosses compared to wild type (P value <0.0002, ****). (TIF) [file pgen.1011774.s005.tif]
