# Supplementary material for: STRIPAK complex defects result in pseudosexual reproduction in Cryptococcus neoformans
Source: PLoS Genet. 2025 Jun 30;21(6):e1011774. doi: 10.1371/journal.pgen.1011774 (PMC12240305; doi:10.1371/journal.pgen.1011774)
Supplement: S1 Table — (DOCX) [file pgen.1011774.s006.docx]

**S1 Table. Strains used in this study.**

| **Strain name** | **Description** | **Source/Reference** |
| --- | --- | --- |
| H99α | Wild-type *MAT*α | [1] |
| KN99**a** | Wild-type *MAT***a** | [2] |
| KN99α | Wild-type *MAT*α | [2] |
| CnLC6683 | Wild-type diploid (KN99**a**/KN99α) | [3] |
| SSH118 | KN99**a** with recombinant mito. genome | [4] |
| PP71 | CnLC6683 *PPH22/pph22*Δ*::NAT* | [3] |
| YSB9100 | H99α *far8*Δ*::NAT* | [3] |
| YSB11129 | YL99**a** *far8*Δ::*NEO* | [3] |
| YSB11132 | YL99**a** *far8*Δ*::NEO* | [3] |
| PP53 | *MAT*α *pph22*Δ*::NAT* | [3] |
| PP55 | *MAT*α *pph22*Δ*::NAT* | [3] |
| PP56 | *MAT***a** *pph22*Δ*::NAT* | [3] |
| PP57 | *MAT***a** *pph22*Δ*::NAT* | [3] |
| PP58 | *MAT***a** *pph22*Δ*::NAT* | [3] |
| PP80 | *MAT*α *pph22*Δ *suppressor* (from PP53) | [3] |
| PP82 | *MAT*α *pph22*Δ *suppressor* (from PP55) | [3] |
| PP83 | *MAT*α *pph22*Δ*-8 suppressor 1* | [3] |
| PP84 | *MAT*α *pph22*Δ*-9 suppressor 1* | [3] |
| MCD16 | H99α *lac1*Δ*::URA5* | [5] |
| PP130 | PP71 *TEF1-PPG1-NEO-4* | This study |
| PP131 | PP71 *TEF1-PPG1-NEO-8* | This study |
| YSB5772 | H99α *ppg1*Δ::*NAT* | [6] |
| YSB5940 | H99α *ppg1*Δ::*NAT* | [6] |
| PP132 | *MAT*α *pph22*Δ*::NAT-2 TEF1-PPG1-NEO* | This study |
| PP133 | *MAT*α *pph22*Δ*::NAT-13 TEF1-PPG1-NEO* | This study |
| PP134 | *MAT***a** *pph22*Δ*::NAT-49 TEF1-PPG1-NEO* | This study |
| PP135 | *MAT***a** *pph22*Δ*::NAT-50 TEF1-PPG1-NEO* | This study |
| PP136 | PP71 *NOP1-GFP-HYG-7* | This study |
| PP137 | PP71 *NOP1-GFP-HYG-15* | This study |
| PP138 | *MAT***a** *pph22*Δ*::NAT-5 NOP1-GFP-HYG* | This study |
| PP139 | *MAT***a** *pph22*Δ*::NAT-7 NOP1-GFP-HYG* | This study |
| PP140 | *MAT***a** *pph22*Δ*::NAT-12 NOP1-GFP-HYG* | This study |
| PP141 | *MAT*α *pph22*Δ*::NAT-8 NOP1-GFP-HYG* | This study |
| YSC46 | KN99**a** *NOP1-GFP-HYG* | This study |
| JOHE18842 | KN99α *NOP1-mCherry-NEO* | Lab stock |
| JOHE18853 | KN99**a** *NOP1-mCherry-NAT* | Lab stock |
| JOHE10493 | KN99**a***::NEO* | Lab stock |
| PP142 | *PPH22/pph22*Δ::*NAT PPG1/ppg1*Δ*::NEO-3* | This study |
| PP143 | *PPH22/pph22*Δ::*NAT PPG1/ppg1*Δ*::NEO-11* | This study |

**References**

1. Perfect JR, Ketabchi N, Cox GM, Ingram CW, Beiser CL. Karyotyping of *Cryptococcus neoformans* as an epidemiological tool. J Clin Microbiol. 1993;31(12):3305-9. doi: 10.1128/jcm.31.12.3305-3309.1993. PubMed PMID: 8308124; PubMed Central PMCID: PMC266409.

2. Nielsen K, Cox GM, Wang P, Toffaletti DL, Perfect JR, Heitman J. Sexual cycle of *Cryptococcus neoformans* var. *grubii* and virulence of congenic **a** and alpha isolates. Infect Immun. 2003;71(9):4831-41. Epub 2003/08/23. doi: 10.1128/IAI.71.9.4831-4841.2003. PubMed PMID: 12933823; PubMed Central PMCID: PMC187335.

3. Peterson PP, Choi JT, Fu C, Cowen LE, Sun S, Bahn YS, et al. The *Cryptococcus neoformans* STRIPAK complex controls genome stability, sexual development, and virulence. PLoS Pathog. 2024;20(11):e1012735. Epub 20241119. doi: 10.1371/journal.ppat.1012735. PubMed PMID: 39561188; PubMed Central PMCID: PMC11614259.

4. Bian Z, Xu Z, Peer A, Choi Y, Priest SJ, Akritidou K, et al. Essential genes encoded by the mating-type locus of the human fungal pathogen *Cryptococcus neoformans*. mBio. 2025:e0022325. Epub 20250225. doi: 10.1128/mbio.00223-25. PubMed PMID: 39998264.

5. Pukkila-Worley R, Gerrald QD, Kraus PR, Boily MJ, Davis MJ, Giles SS, et al. Transcriptional network of multiple capsule and melanin genes governed by the *Cryptococcus neoformans* cyclic AMP cascade. Eukaryot Cell. 2005;4(1):190-201. Epub 2005/01/12. doi: 10.1128/EC.4.1.190-201.2005. PubMed PMID: 15643074; PubMed Central PMCID: PMC544166.

6. Jin JH, Lee KT, Hong J, Lee D, Jang EH, Kim JY, et al. Genome-wide functional analysis of phosphatases in the pathogenic fungus *Cryptococcus neoformans*. Nature Communications. 2020;11(1):4212. Epub 2020/08/26. doi: 10.1038/s41467-020-18028-0. PubMed PMID: 32839469; PubMed Central PMCID: PMC7445287.
