# Supplementary material for: STRIPAK complex defects result in pseudosexual reproduction in Cryptococcus neoformans
Source: PLoS Genet. 2025 Jun 30;21(6):e1011774. doi: 10.1371/journal.pgen.1011774 (PMC12240305; doi:10.1371/journal.pgen.1011774)
Supplement: S2 Table — (DOCX) [file pgen.1011774.s007.docx]

**S2 Table. Primers used in this study.**

| **Primer #** | **Sequence** | **Purpose** |
| --- | --- | --- |
| JOHE53750 | CTAACTCTACTACACCTCACGGCA | *MAT***a** genotyping (*STE20***a**) |
| JOHE52751 | CGCACTGCAAAATAGATAAGTCTG |  |
| JOHE52752 | GGCTGCAATCACAGCACCTTAC | *MAT*α genotyping (*STE20*α) |
| JOHE52753 | CTTCATGACATCACTCCCCTAT |  |
| JOHE52754 | TGGTGGTGGTGACCCAGTTCT | Mitochondria genotyping (*COX1)* |
| JOHE52755 | CCGAAGATCTTAGGTGCCCA |  |
| M13F | GTAAAACGACGGCCAG | To amplify drug resistance cassettes |
| M13R | CAGGAAACAGCTATGAC | To amplify drug resistance cassettes |
| JOHE52463 | CTGGCGGAGGATAGAAGC | *ACT1* promoter reverse primer |
| JOHE52464 | GCGAATTCGAGACAGACATCG | *TRP1* terminator forward primer |
| JOHE56046 | AATTGGGTACCGGGCCCCCCCCAAGATTGTGGCTACTAT | *TEF1-PPG1-NEO* overexpression construct (cloned into pSDMA57) |
| JOHE56047 | GAAGTTTTCTGTGGAGA |  |
| JOHE56048 | CGATCTCCACAGAAAACTTCATGGCACCGTTCGACCT |  |
| JOHE56049 | GCGGCCGCTCTAGAAGAGAAAGTATTCGATTTG |  |
| JOHE56050 | CACCGGCAGGGTATACTGTTAAGGGCCAATGAAGCACGCTGTTTTAGAGCTAGAAATAGCAAG | Safe haven gRNA |
| JOHE50175 | ACTGGTGAGTACTCAACCAAG | Safe haven screening primers |
| JOHE50176 | GGGTATGCCACAGATGCAGAT |  |
| JOHE50177 | TTGGATCCTCAATTGTCTCCT |  |
| JOHE50651 | GTCTTCTCCTTGTCTACAGG | To generate *ppg1::NEO* deletion construct |
| JOHE50652 | CTGGCCGTCGTTTTACTCCATTAAGCAAAGAGGGGG |  |
| JOHE50653 | GTCATAGCTGTTTCCTGATACAGTACCCTGCATATCG |  |
| JOHE50654 | CATGTTCTCTTTTCGCTTCC |  |
| JOHE50655 | CACCGGCAGGGTATACTGTTGAACGCATCCAGCTTATTCGGTTTTAGAGCTAGAAATAGCAAG |  |
| JOHE50656 | TGTACCTCATCGGCCAAAAT | *ppg1::NEO* screening primers |
| JOHE50657 | CTTCTTCTCACCCATGACCA |  |
| JOHE54049 | CCACAACACATCTATCACGCGGCCGCATGGCTTTCGGTGACAGAG | GFP-tagging of *NOP1* (cloned into YSCE5) |
| JOHE54256 | ATAGAGCCACCGCCACCTGCGGCCGCAGTGTGTCGTTGGTATATGC |  |
