# Supplementary material for: STRIPAK complex defects result in pseudosexual reproduction in Cryptococcus neoformans
Source: PLoS Genet. 2025 Jun 30;21(6):e1011774. doi: 10.1371/journal.pgen.1011774 (PMC12240305; doi:10.1371/journal.pgen.1011774)
Supplement: S3 Table — (DOCX) [file pgen.1011774.s008.docx]

**S3 Table. RNA sequencing analysis of *pph22*Δ and *far8*Δ mutants.** The table depicts the log_2_ fold change and statistical analysis of differentially expressed genes in the indicated deletion mutants compared to the isogenic wild-type control strain. Functional categories were assigned to the selected genes based on their annotations on FungiDB.

| **Gene ID** | ***pph22* vs WT log2 FC** | **p-value** | **p-adj. value** | ***far8* vs WT log2 FC** | **p-value** | **p-adj. value** | **Functional category** |
| --- | --- | --- | --- | --- | --- | --- | --- |
| CNAG_02183 | -1.650501141 | 2.2E-08 | 4.62E-07 | 0.494706827 | 0.000205564 | 0.000419556 | Cell Cycle Regulation |
| CNAG_00183 | -1.154341123 | 0.012559 | 0.0485608 | -1.174090607 | 2.50E-10 | 9.24E-10 | Cell Cycle Regulation |
| CNAG_02541 | -1.025503826 | 0.001708 | 0.0098203 | -0.195684589 | 0.108932284 | 0.146648433 | Cell Cycle Regulation |
| CNAG_03137 | -0.875243953 | 0.019553 | 0.0682548 | 0.183182565 | 0.200968648 | 0.254052976 | Cell Cycle Regulation |
| CNAG_01633 | 0.855824504 | 0.008155 | 0.0339065 | 0.40808207 | 1.53465E-06 | 3.97183E-06 | Cell Cycle Regulation |
| CNAG_03932 | 0.973948835 | 0.034719 | 0.106092 | 0.484189096 | 1.04965E-06 | 2.76568E-06 | Cell Cycle Regulation |
| CNAG_06086 | 1.019597487 | 0.036578 | 0.1105124 | -0.028247174 | 0.806806113 | 0.839421679 | Cell Cycle Regulation |
| CNAG_03171 | 1.02119697 | 0.004396 | 0.0209839 | 0.766827467 | 1.12178E-08 | 3.56474E-08 | Cell Cycle Regulation |
| CNAG_05549 | 1.225993463 | 0.001078 | 0.0066891 | 0.28335688 | 0.000348066 | 0.000687938 | Cell Cycle Regulation |
| CNAG_00437 | 1.24075671 | 0.087753 | 0.2115295 | 0.698726481 | 2.77245E-08 | 8.48266E-08 | Cell Cycle Regulation |
| CNAG_06031 | -2.086050591 | 0.036778 | 0.1109723 | -0.065564478 | 0.678522738 | 0.731305526 | Cell Wall |
| CNAG_02867 | 1.755569334 | 0.005126 | 0.0235467 | 0.214217098 | 0.066716862 | 0.093717012 | Cell Wall |
| CNAG_03765 | -1.048433038 | 0.00198 | 0.0110763 | -1.284579022 | 1.00E-23 | 1.01E-22 | Cell Wall |
| CNAG_01523 | -0.817893778 | 0.02116 | 0.0726928 | -0.351208788 | 0.000238284 | 0.000481871 | Cell Wall |
| CNAG_05292 | -0.80013919 | 0.01645 | 0.060037 | -1.565081043 | 7.55E-22 | 6.77E-21 | Cell Wall |
| CNAG_00393 | -0.794074577 | 0.035381 | 0.1077472 | -2.748519615 | 1.64E-80 | 1.89E-78 | Cell Wall |
| CNAG_04245 | 0.745863452 | 0.036459 | 0.1102963 | 0.005655716 | 0.961357831 | 0.968229664 | Cell Wall |
| CNAG_02860 | 0.766543369 | 0.01609 | 0.0592016 | -0.252767974 | 0.006914166 | 0.01142466 | Cell Wall |
| CNAG_05818 | 0.918680436 | 0.004071 | 0.0197553 | 0.426592007 | 0.000503521 | 0.000973888 | Cell Wall |
| CNAG_06336 | 1.007282559 | 0.030238 | 0.0956313 | 0.250765384 | 0.005077705 | 0.008555119 | Cell Wall |
| CNAG_02511 | 1.020370389 | 0.153967 | 0.3143435 | 0.666374011 | 1.451E-06 | 3.76353E-06 | Cell Wall |
| CNAG_06845 | 1.0341206 | 0.005203 | 0.0238245 | 0.559726624 | 3.51752E-07 | 9.76007E-07 | Cell Wall |
| CNAG_04282 | 1.039929061 | 0.057994 | 0.1564056 | 0.311976724 | 0.001993679 | 0.003580352 | Cell Wall |
| CNAG_07499 | 1.089856571 | 0.002474 | 0.0132826 | 0.132720101 | 0.171209519 | 0.21970743 | Cell Wall |
| CNAG_05799 | 1.101399797 | 6.77E-05 | 0.0006249 | 0.292640263 | 0.006218408 | 0.010344932 | Cell Wall |
| CNAG_06658 | 1.131652683 | 0.006239 | 0.027431 | 1.922902176 | 5.83E-82 | 7.23E-80 | Cell Wall |
| CNAG_01941 | 1.216209719 | 5.17E-05 | 0.0004934 | 0.782472873 | 9.69E-14 | 4.83E-13 | Cell Wall |
| CNAG_00546 | 1.243633204 | 0.002042 | 0.0113392 | -0.31522391 | 0.000442111 | 0.000861728 | Cell Wall |
| CNAG_05458 | 1.281143699 | 0.077849 | 0.1936216 | 2.675898515 | 3.51E-100 | 8.83E-98 | Cell Wall |
| CNAG_03099 | 1.295737824 | 3.92E-06 | 0.0000513 | 0.907046876 | 1.21E-12 | 5.55E-12 | Cell Wall |
| CNAG_02670 | 1.305672194 | 4.52E-05 | 0.0004417 | 0.854649357 | 5.29E-15 | 2.89E-14 | Cell Wall |
| CNAG_06898 | 1.354950876 | 0.013106 | 0.0502036 | 0.74998219 | 2.75E-23 | 2.66E-22 | Cell Wall |
| CNAG_04859 | 1.399952498 | 0.001502 | 0.0088814 | 0.555050645 | 6.50304E-06 | 1.58208E-05 | Cell Wall |
| CNAG_05866 | 1.405892439 | 0.002366 | 0.0127932 | 1.105117737 | 3.65E-20 | 2.91E-19 | Cell Wall |
| CNAG_03234 | 1.429513358 | 0.00025 | 0.0019139 | 0.824749776 | 0.000140007 | 0.000291042 | Cell Wall |
| CNAG_01938 | 1.438697017 | 2.1E-06 | 0.0000293 | -0.165091582 | 0.04372572 | 0.063381327 | Cell Wall |
| CNAG_02217 | 1.581291328 | 0.000804 | 0.0052586 | 2.088522324 | 9.59E-30 | 1.38E-28 | Cell Wall |
| CNAG_03326 | 1.768800679 | 6.67E-06 | 0.0000828 | 0.409924833 | 0.007464747 | 0.012275854 | Cell Wall |
| CNAG_07736 | 1.92111591 | 1.08E-13 | 7.06E-12 | 0.203467366 | 0.287155639 | 0.349403517 | Cell Wall |
| CNAG_03130 | 2.394181686 | 1.53E-08 | 3.42E-07 | 0.315741491 | 0.011178923 | 0.017888428 | Cell Wall |
| CNAG_00939 | 2.818368995 | 8.79E-14 | 6E-12 | -0.804458982 | 0.252502641 | 0.311895164 | Cell Wall |
| CNAG_00373 | 3.506568035 | 7.06E-16 | 7.68E-14 | 0.902406952 | 2.01E-19 | 1.53E-18 | Cell Wall |
| CNAG_02225 | 3.682149894 | 4.51E-10 | 1.33E-08 | 0.271599025 | 0.060406475 | 0.085532112 | Cell Wall |
| CNAG_01013 | -1.556853863 | 0.000134 | 0.001132 | -1.294622849 | 9.73E-16 | 5.65E-15 | Chromatin Structure & Transcription Regulation |
| CNAG_00561 | -1.228714024 | 0.001393 | 0.0083359 | -0.292701072 | 0.001166966 | 0.002164434 | Chromatin Structure & Transcription Regulation |
| CNAG_07712 | -1.175947955 | 5.89E-06 | 0.0000745 | -0.378243674 | 0.00234774 | 0.00417114 | Chromatin Structure & Transcription Regulation |
| CNAG_07464 | -1.127219776 | 0.007604 | 0.0319965 | 0.595022766 | 3.96E-11 | 1.58E-10 | Chromatin Structure & Transcription Regulation |
| CNAG_04450 | -1.104014937 | 1.32E-05 | 0.0001497 | -0.525402314 | 1.24616E-09 | 4.31278E-09 | Chromatin Structure & Transcription Regulation |
| CNAG_04381 | -1.062782513 | 0.081283 | 0.2000203 | -0.645967109 | 1.03E-11 | 4.31E-11 | Chromatin Structure & Transcription Regulation |
| CNAG_06544 | -1.061830473 | 3.15E-06 | 0.0000421 | -0.152051399 | 0.063140516 | 0.089160371 | Chromatin Structure & Transcription Regulation |
| CNAG_04828 | -1.039005185 | 0.003901 | 0.0191483 | 0.791386942 | 7.63E-15 | 4.11E-14 | Chromatin Structure & Transcription Regulation |
| CNAG_06746 | -1.007362443 | 1.54E-05 | 0.0001722 | -0.280821573 | 0.002147138 | 0.003834405 | Chromatin Structure & Transcription Regulation |
| CNAG_01648 | -0.962401678 | 0.002266 | 0.0123942 | -0.095086663 | 0.383185596 | 0.449854804 | Chromatin Structure & Transcription Regulation |
| CNAG_06745 | -0.927982797 | 1.76E-07 | 3.15E-06 | -0.068596887 | 0.45815916 | 0.524972722 | Chromatin Structure & Transcription Regulation |
| CNAG_06747 | -0.910887676 | 0.000909 | 0.0057881 | -0.230938146 | 0.021713824 | 0.033138795 | Chromatin Structure & Transcription Regulation |
| CNAG_01018 | -0.869031763 | 0.002226 | 0.0122324 | -0.546695009 | 1.63E-16 | 1.00E-15 | Chromatin Structure & Transcription Regulation |
| CNAG_05166 | -0.773857305 | 0.001998 | 0.0111566 | -0.067082196 | 0.541494785 | 0.605411534 | Chromatin Structure & Transcription Regulation |
| CNAG_01438 | -0.794797871 | 0.0595 | 0.1592463 | 0.270218405 | 0.005761478 | 0.009630661 | Chromatin Structure & Transcription Regulation |
| CNAG_05404 | 1.004512986 | 0.00274 | 0.0144793 | 0.006828463 | 0.938110953 | 0.948917028 | Chromatin Structure & Transcription Regulation |
| CNAG_06068 | 1.02027267 | 0.001352 | 0.0081336 | -0.185752465 | 0.023553989 | 0.03568513 | Chromatin Structure & Transcription Regulation |
| CNAG_02749 | 1.099621664 | 0.000176 | 0.0014222 | 0.754407852 | 7.39E-16 | 4.33E-15 | Chromatin Structure & Transcription Regulation |
| CNAG_00360 | 1.100084871 | 0.002481 | 0.0133134 | -0.650650777 | 1.43008E-07 | 4.10932E-07 | Chromatin Structure & Transcription Regulation |
| CNAG_07866 | 1.274027016 | 0.226407 | 0.4101945 | 0.004540975 | 0.974595319 | 0.979070802 | Chromatin Structure & Transcription Regulation |
| CNAG_03129 | 1.291631265 | 0.034568 | 0.1058612 | 1.906421337 | 8.33E-30 | 1.21E-28 | Chromatin Structure & Transcription Regulation |
| CNAG_05276 | 1.524833822 | 6.38E-07 | 0.00001 | -0.292130469 | 0.005264978 | 0.008845545 | Chromatin Structure & Transcription Regulation |
| CNAG_05392 | 1.558796879 | 0.087336 | 0.2108177 | 1.465593521 | 3.33E-17 | 2.15E-16 | Chromatin Structure & Transcription Regulation |
| CNAG_01306 | 1.651649664 | 0.002155 | 0.0119216 | 0.327727187 | 0.00100126 | 0.001870443 | Chromatin Structure & Transcription Regulation |
| CNAG_00068 | 1.738488383 | 0.014735 | 0.0552586 | 1.004914462 | 3.04E-22 | 2.78E-21 | Chromatin Structure & Transcription Regulation |
| CNAG_00330 | 1.856858418 | 0.000134 | 0.0011356 | 1.250053395 | 1.59E-24 | 1.68E-23 | Chromatin Structure & Transcription Regulation |
| CNAG_03003 | 1.896284993 | 5.89E-14 | 4.17E-12 | -0.016859894 | 0.853771527 | 0.880025134 | Chromatin Structure & Transcription Regulation |
| CNAG_00665 | -1.086864385 | 0.00224 | 0.0122744 | -0.434319073 | 1.9664E-07 | 5.58098E-07 | Cell cycle & cell division |
| CNAG_03787 | -0.979950994 | 0.000482 | 0.0033872 | -0.2331374 | 0.010587625 | 0.017011002 | Cell cycle & cell division |
| CNAG_02267 | -0.951779163 | 0.00183 | 0.0104047 | -0.048846581 | 0.686030067 | 0.73748454 | Cell cycle & cell division |
| CNAG_04948 | -0.903451201 | 0.007527 | 0.0317124 | -0.816324033 | 2.28E-15 | 1.29E-14 | Cell cycle & cell division |
| CNAG_01391 | -0.820619716 | 0.010784 | 0.0426941 | -0.72772754 | 7.26E-13 | 3.37E-12 | Cell cycle & cell division |
| CNAG_01362 | -0.706857772 | 0.027315 | 0.0881821 | -1.212897717 | 1.67E-66 | 1.35E-64 | Cell cycle & cell division |
| CNAG_01638 | 0.705017325 | 0.163059 | 0.3278136 | 0.781883577 | 3.76E-11 | 1.51E-10 | Cell cycle & cell division |
| CNAG_00423 | 0.985313113 | 0.215162 | 0.3972614 | 1.323772465 | 2.32E-19 | 1.76E-18 | Cell cycle & cell division |
| CNAG_00545 | 1.019617959 | 0.003097 | 0.0159454 | 0.692742864 | 2.13E-10 | 7.93E-10 | Cell cycle & cell division |
| CNAG_05558 | 1.077650072 | 0.041163 | 0.1202921 | 1.191485506 | 8.67E-33 | 1.46E-31 | Cell cycle & cell division |
| CNAG_04055 | 1.090034523 | 0.017234 | 0.0622837 | 0.811135832 | 1.42E-15 | 8.16E-15 | Cell cycle & cell division |
| CNAG_01325 | 1.136502026 | 0.100969 | 0.2337595 | 0.162547193 | 0.228073357 | 0.284851835 | Cell cycle & cell division |
| CNAG_03835 | 1.701674329 | 0.271924 | 0.4635688 | 1.56496705 | 6.50E-24 | 6.60E-23 | Cell cycle & cell division |
| CNAG_04007 | 1.799490264 | 6.61E-07 | 0.0000103 | 0.883889111 | 0.009563719 | 0.015449514 | Cell cycle & cell division |
| CNAG_02082 | 2.23630347 | 0.040828 | 0.1194654 | 2.137965381 | 4.97E-29 | 6.87E-28 | Cell cycle & cell division |
| CNAG_04648 | 1.30192815 | 5.19E-05 | 0.0004945 | 0.215157638 | 0.029655692 | 0.044196444 | Cell cycle & cell division |
| CNAG_05825 | -1.60696027 | 1.37E-05 | 0.0001554 | -0.18058813 | 0.062918289 | 0.088906976 | DNA Replication & Genome Integrity |
| CNAG_00772 | -1.351878079 | 0.000517 | 0.0035945 | -0.507227831 | 4.69276E-07 | 1.28622E-06 | DNA Replication & Genome Integrity |
| CNAG_02467 | -1.333833317 | 0.000433 | 0.0030913 | -1.996042329 | 7.02E-58 | 3.94E-56 | DNA Replication & Genome Integrity |
| CNAG_03771 | -1.331116591 | 0.000482 | 0.0033872 | -0.041610874 | 0.672224037 | 0.725928607 | DNA Replication & Genome Integrity |
| CNAG_06142 | -1.324819845 | 0.000499 | 0.0034833 | 0.420503741 | 1.37229E-06 | 3.56272E-06 | DNA Replication & Genome Integrity |
| CNAG_00705 | -1.050587675 | 0.000557 | 0.0038299 | 0.131977179 | 0.243268354 | 0.301519283 | DNA Replication & Genome Integrity |
| CNAG_05531 | -0.927330671 | 0.008314 | 0.034421 | -0.034313324 | 0.715731604 | 0.763196104 | DNA Replication & Genome Integrity |
| CNAG_00108 | -0.823402114 | 0.004282 | 0.0205668 | -1.678106832 | 1.66E-51 | 6.73E-50 | DNA Replication & Genome Integrity |
| CNAG_01916 | -0.727295029 | 0.036127 | 0.1096254 | -0.083107961 | 0.320783891 | 0.384524368 | DNA Replication & Genome Integrity |
| CNAG_01163 | 0.728010616 | 0.11981 | 0.263703 | 0.953181204 | 5.44E-30 | 7.94E-29 | DNA Replication & Genome Integrity |
| CNAG_03449 | 0.862600099 | 0.054754 | 0.1497095 | 0.766964333 | 7.53E-18 | 5.12E-17 | DNA Replication & Genome Integrity |
| CNAG_05537 | 1.008367687 | 0.016028 | 0.0590269 | 0.828417182 | 8.73175E-09 | 2.79719E-08 | DNA Replication & Genome Integrity |
| CNAG_02771 | 1.021866324 | 0.007682 | 0.0322455 | 0.786245892 | 4.10E-14 | 2.10E-13 | DNA Replication & Genome Integrity |
| CNAG_02480 | 1.075231232 | 0.034453 | 0.1056037 | 0.351061377 | 0.011003306 | 0.017620976 | DNA Replication & Genome Integrity |
| CNAG_05177 | 1.085029436 | 0.04067 | 0.1192035 | 0.722389736 | 2.21432E-06 | 5.63959E-06 | DNA Replication & Genome Integrity |
| CNAG_00720 | 1.09527208 | 0.019129 | 0.0671602 | 1.455033066 | 6.88E-31 | 1.05E-29 | DNA Replication & Genome Integrity |
| CNAG_01792 | 1.139005054 | 0.000324 | 0.0024096 | 1.103834917 | 1.76E-20 | 1.43E-19 | DNA Replication & Genome Integrity |
| CNAG_03813 | 1.175544658 | 0.011717 | 0.0458933 | 0.654297586 | 8.80E-11 | 3.40E-10 | DNA Replication & Genome Integrity |
| CNAG_02654 | 1.196489282 | 6.83E-05 | 0.000628 | 0.057299237 | 0.542102478 | 0.605956421 | DNA Replication & Genome Integrity |
| CNAG_04682 | 1.226847144 | 0.053647 | 0.147959 | 1.154278165 | 2.88E-26 | 3.36E-25 | DNA Replication & Genome Integrity |
| CNAG_03374 | 1.369213227 | 0.000918 | 0.005834 | 1.435309953 | 7.46E-21 | 6.23E-20 | DNA Replication & Genome Integrity |
| CNAG_03160 | 1.464753754 | 1.1E-06 | 0.0000165 | 1.199843623 | 2.89E-13 | 1.38E-12 | DNA Replication & Genome Integrity |
| CNAG_03795 | 1.476155166 | 1.23E-10 | 4.03E-09 | 0.327016661 | 3.17457E-05 | 7.11711E-05 | DNA Replication & Genome Integrity |
| CNAG_04052 | 1.482391033 | 0.001522 | 0.0089616 | 0.461339733 | 0.030185973 | 0.044962551 | DNA Replication & Genome Integrity |
| CNAG_07766 | 1.496083899 | 0.000489 | 0.003426 | 0.286098167 | 0.041328043 | 0.060189212 | DNA Replication & Genome Integrity |
| CNAG_00178 | 1.639327729 | 5.36E-05 | 0.0005081 | -0.146905506 | 0.125695573 | 0.166918099 | DNA Replication & Genome Integrity |
| CNAG_01891 | 1.774241237 | 8.51E-09 | 1.97E-07 | 1.258064502 | 8.74E-38 | 1.81E-36 | DNA Replication & Genome Integrity |
| CNAG_00384 | 1.84156047 | 0.001352 | 0.0081336 | 1.735133101 | 4.82E-12 | 2.09E-11 | DNA Replication & Genome Integrity |
| CNAG_02563 | 1.844926284 | 7.55E-07 | 0.0000116 | 0.80017153 | 1.43574E-07 | 4.12415E-07 | DNA Replication & Genome Integrity |
| CNAG_03330 | 1.853480847 | 0.018873 | 0.0667192 | 0.772276343 | 2.97E-18 | 2.07E-17 | DNA Replication & Genome Integrity |
| CNAG_04787 | 1.983043394 | 1.68E-09 | 4.51E-08 | 0.560113732 | 1.99971E-07 | 5.67165E-07 | DNA Replication & Genome Integrity |
| CNAG_00419 | 1.991572463 | 1.3E-17 | 2.11E-15 | 0.172390137 | 0.112634002 | 0.151142286 | DNA Replication & Genome Integrity |
| CNAG_00299 | 2.023026524 | 3.85E-09 | 9.64E-08 | 0.358030012 | 0.000354477 | 0.000699946 | DNA Replication & Genome Integrity |
| CNAG_06525 | -1.552603236 | 0.405318 | 0.6033354 | 0.604932695 | 0.001957453 | 0.003520716 | Nuclear Organization |
| CNAG_02404 | -1.51636751 | 8.26E-10 | 2.36E-08 | -0.687487344 | 8.69211E-09 | 2.78556E-08 | Nuclear Organization |
| CNAG_01361 | -1.434949226 | 2.05E-06 | 0.0000287 | 0.911411794 | 6.02509E-07 | 1.62886E-06 | Nuclear Organization |
| CNAG_05596 | -1.39434381 | 0.000566 | 0.003884 | 1.131810297 | 3.78E-19 | 2.80E-18 | Nuclear Organization |
| CNAG_05884 | -1.226892736 | 1.45E-05 | 0.0001632 | -0.071924384 | 0.669904429 | 0.72370572 | Nuclear Organization |
| CNAG_00413 | -1.203888635 | 3.76E-05 | 0.0003808 | 0.696962762 | 6.7789E-07 | 1.82195E-06 | Nuclear Organization |
| CNAG_02990 | -1.163149101 | 0.000652 | 0.00439 | -0.428977988 | 1.98425E-08 | 6.16869E-08 | Nuclear Organization |
| CNAG_02720 | -1.072650635 | 0.000092 | 0.0008146 | -0.727321406 | 4.79E-21 | 4.05E-20 | Nuclear Organization |
| CNAG_02257 | -1.057108799 | 0.000737 | 0.0048888 | 0.091677964 | 0.298124179 | 0.36105861 | Nuclear Organization |
| CNAG_06224 | -1.020106778 | 0.020612 | 0.0712712 | -0.448507912 | 9.68226E-06 | 2.30693E-05 | Nuclear Organization |
| CNAG_01732 | -0.962222939 | 0.003289 | 0.0167677 | -0.537975059 | 7.80455E-05 | 0.000167344 | Nuclear Organization |
| CNAG_02816 | -0.884978607 | 0.004085 | 0.0197956 | -0.173038056 | 0.023496424 | 0.035610897 | Nuclear Organization |
| CNAG_07598 | -0.844271861 | 0.008439 | 0.0347735 | 0.07009576 | 0.50039363 | 0.566649263 | Nuclear Organization |
| CNAG_02265 | -0.804060486 | 0.013849 | 0.0525478 | -0.864835986 | 7.31E-23 | 6.95E-22 | Nuclear Organization |
| CNAG_01053 | -0.705968824 | 0.019684 | 0.0686768 | -0.399962838 | 7.79237E-06 | 1.87599E-05 | Nuclear Organization |
| CNAG_06280 | 1.139863465 | 0.004922 | 0.0227923 | -0.508038499 | 2.60438E-09 | 8.78662E-09 | Nuclear Organization |
| CNAG_02008 | 1.50433676 | 0.000226 | 0.0017505 | 0.01642846 | 0.83973407 | 0.8680315 | Nuclear Organization |
| CNAG_02268 | 1.891096784 | 1.78E-07 | 3.18E-06 | 0.41033962 | 0.000494143 | 0.000956862 | Nuclear Organization |
| CNAG_06611 | -1.271453147 | 5.06E-05 | 0.0004846 | -0.277377722 | 0.002038826 | 0.003653537 | RNA Processing & Ribosome Biogenesis |
| CNAG_02022 | -1.12963425 | 0.006629 | 0.0287442 | 0.696298515 | 1.93E-10 | 7.23E-10 | RNA Processing & Ribosome Biogenesis |
| CNAG_07374 | 1.267156248 | 0.00283 | 0.0148538 | -0.152131525 | 0.375448276 | 0.442375598 | RNA Processing & Ribosome Biogenesis |
| CNAG_03521 | 1.493191944 | 0.070466 | 0.1806803 | 1.309146383 | 6.34E-17 | 4.02E-16 | RNA Processing & Ribosome Biogenesis |
| CNAG_02418 | -2.415431568 | 1.1E-18 | 2.65E-16 | -0.812495365 | 1.15E-11 | 4.81E-11 | RNA Processing & Ribosome Biogenesis |
| CNAG_05199 | -1.906015979 | 2.97E-13 | 1.67E-11 | -0.276078098 | 0.004985407 | 0.008411546 | RNA Processing & Ribosome Biogenesis |
| CNAG_00785 | -1.713228909 | 8.44E-11 | 2.85E-09 | 0.008226541 | 0.92788275 | 0.940898914 | RNA Processing & Ribosome Biogenesis |
| CNAG_04179 | -1.701012241 | 2.53E-11 | 9.57E-10 | -0.553801453 | 3.95E-11 | 1.58E-10 | RNA Processing & Ribosome Biogenesis |
| CNAG_05827 | -1.593507089 | 3.36E-09 | 8.57E-08 | -0.448921086 | 6.74247E-08 | 1.99467E-07 | RNA Processing & Ribosome Biogenesis |
| CNAG_03641 | -1.53636584 | 1.56E-08 | 3.46E-07 | -0.396024707 | 4.36615E-07 | 1.20185E-06 | RNA Processing & Ribosome Biogenesis |
| CNAG_01428 | -1.531537768 | 1.2E-12 | 5.88E-11 | -0.902015167 | 3.03E-19 | 2.27E-18 | RNA Processing & Ribosome Biogenesis |
| CNAG_04441 | -1.510202695 | 8.62E-13 | 4.35E-11 | -0.297325267 | 0.001286333 | 0.002370492 | RNA Processing & Ribosome Biogenesis |
| CNAG_07362 | -1.450014589 | 5.3E-08 | 1.05E-06 | 0.259851161 | 0.002295286 | 0.004084056 | RNA Processing & Ribosome Biogenesis |
| CNAG_01111 | -1.422236454 | 5.03E-05 | 0.0004825 | -0.766809052 | 1.08E-21 | 9.53E-21 | RNA Processing & Ribosome Biogenesis |
| CNAG_06755 | -1.404222959 | 9.4E-06 | 0.0001109 | -0.373736508 | 2.77086E-05 | 6.24909E-05 | RNA Processing & Ribosome Biogenesis |
| CNAG_00238 | -1.397020484 | 7.18E-09 | 1.69E-07 | -0.128022956 | 0.134955525 | 0.17793581 | RNA Processing & Ribosome Biogenesis |
| CNAG_06906 | -1.39091693 | 1.31E-08 | 2.97E-07 | -0.721543672 | 1.49E-17 | 9.96E-17 | RNA Processing & Ribosome Biogenesis |
| CNAG_00741 | -1.382421848 | 2.46E-07 | 4.23E-06 | -0.008146577 | 0.933980273 | 0.945812889 | RNA Processing & Ribosome Biogenesis |
| CNAG_07628 | -1.369818518 | 3.91E-08 | 7.94E-07 | 0.013716314 | 0.895691569 | 0.915177484 | RNA Processing & Ribosome Biogenesis |
| CNAG_01537 | -1.367491886 | 0.000017 | 0.0001892 | -0.401203281 | 4.99162E-07 | 1.3614E-06 | RNA Processing & Ribosome Biogenesis |
| CNAG_04260 | -1.364134777 | 0.001167 | 0.0071692 | -0.335625117 | 7.71869E-05 | 0.000165631 | RNA Processing & Ribosome Biogenesis |
| CNAG_06123 | -1.363061683 | 4.94E-06 | 0.0000634 | -0.76374362 | 7.53E-19 | 5.47E-18 | RNA Processing & Ribosome Biogenesis |
| CNAG_05105 | -1.360755308 | 4.94E-05 | 0.000475 | -0.36457604 | 0.000963267 | 0.001803116 | RNA Processing & Ribosome Biogenesis |
| CNAG_05900 | -1.346688056 | 7.41E-07 | 0.0000114 | -0.258718793 | 0.0006432 | 0.001231159 | RNA Processing & Ribosome Biogenesis |
| CNAG_03263 | -1.332182686 | 2.66E-07 | 4.53E-06 | -0.324095796 | 0.020187923 | 0.030980654 | RNA Processing & Ribosome Biogenesis |
| CNAG_00386 | -1.331725773 | 1.25E-09 | 3.49E-08 | 0.025694428 | 0.727236597 | 0.773182994 | RNA Processing & Ribosome Biogenesis |
| CNAG_00084 | -1.318261174 | 4.49E-05 | 0.0004395 | 0.070447454 | 0.381870173 | 0.448563903 | RNA Processing & Ribosome Biogenesis |
| CNAG_02657 | -1.316565235 | 0.000001 | 0.0000151 | -0.424306329 | 4.47894E-08 | 1.34709E-07 | RNA Processing & Ribosome Biogenesis |
| CNAG_07778 | -1.306751735 | 7.26E-06 | 0.0000891 | -0.325273258 | 1.65851E-06 | 4.27775E-06 | RNA Processing & Ribosome Biogenesis |
| CNAG_01548 | -1.248875647 | 3.86E-05 | 0.0003862 | -0.257182771 | 0.001196352 | 0.002213998 | RNA Processing & Ribosome Biogenesis |
| CNAG_02128 | -1.248013026 | 9.52E-06 | 0.0001117 | -0.007105235 | 0.946990066 | 0.956656753 | RNA Processing & Ribosome Biogenesis |
| CNAG_00822 | -1.244672396 | 7.86E-05 | 0.0007105 | 0.432087258 | 4.06131E-07 | 1.12053E-06 | RNA Processing & Ribosome Biogenesis |
| CNAG_00781 | -1.241092132 | 0.017336 | 0.0624606 | -0.196257723 | 0.080333383 | 0.111082915 | RNA Processing & Ribosome Biogenesis |
| CNAG_04082 | -1.234662239 | 1.8E-07 | 3.21E-06 | -0.040304822 | 0.603830243 | 0.663184148 | RNA Processing & Ribosome Biogenesis |
| CNAG_07651 | -1.233640218 | 0.189974 | 0.3644574 | 0.290818336 | 0.083200851 | 0.114831571 | RNA Processing & Ribosome Biogenesis |
| CNAG_06585 | -1.230827762 | 2.21E-07 | 3.87E-06 | -0.091497775 | 0.321559009 | 0.385286833 | RNA Processing & Ribosome Biogenesis |
| CNAG_00305 | -1.202535317 | 7.51E-05 | 0.000685 | 0.213919629 | 0.016739692 | 0.026025259 | RNA Processing & Ribosome Biogenesis |
| CNAG_01198 | -1.189969295 | 0.004683 | 0.0219524 | -0.466595174 | 9.19606E-05 | 0.000194868 | RNA Processing & Ribosome Biogenesis |
| CNAG_04604 | -1.182056132 | 0.000035 | 0.000356 | -0.383441813 | 8.9187E-05 | 0.000189522 | RNA Processing & Ribosome Biogenesis |
| CNAG_01435 | -1.173410859 | 6.48E-06 | 0.0000805 | -0.811499426 | 3.53E-16 | 2.12E-15 | RNA Processing & Ribosome Biogenesis |
| CNAG_03457 | -1.172659863 | 4.31E-05 | 0.0004247 | -0.357623681 | 0.003823722 | 0.006579714 | RNA Processing & Ribosome Biogenesis |
| CNAG_02212 | -1.17196644 | 4.3E-09 | 1.06E-07 | -0.013585455 | 0.890083443 | 0.910566535 | RNA Processing & Ribosome Biogenesis |
| CNAG_05366 | -1.141713695 | 9.72E-06 | 0.0001138 | -0.564701915 | 8.88E-13 | 4.11E-12 | RNA Processing & Ribosome Biogenesis |
| CNAG_05978 | -1.135464427 | 3.71E-05 | 0.0003758 | -0.545099035 | 5.36448E-09 | 1.7564E-08 | RNA Processing & Ribosome Biogenesis |
| CNAG_06563 | -1.134863153 | 0.000382 | 0.0027593 | -0.220500227 | 0.002784797 | 0.004898438 | RNA Processing & Ribosome Biogenesis |
| CNAG_01103 | -1.13447906 | 0.021319 | 0.0731711 | -0.175768556 | 0.073119548 | 0.101918645 | RNA Processing & Ribosome Biogenesis |
| CNAG_03246 | -1.133058553 | 0.09221 | 0.2191649 | 0.808989781 | 2.64E-10 | 9.74E-10 | RNA Processing & Ribosome Biogenesis |
| CNAG_05976 | -1.128505533 | 3.28E-05 | 0.0003391 | 0.230344211 | 0.042935859 | 0.062356009 | RNA Processing & Ribosome Biogenesis |
| CNAG_05826 | -1.127086819 | 0.00015 | 0.0012451 | 0.003450204 | 0.963919276 | 0.969986799 | RNA Processing & Ribosome Biogenesis |
| CNAG_07511 | -1.123033618 | 0.000426 | 0.00305 | -1.030750114 | 1.30E-37 | 2.66E-36 | RNA Processing & Ribosome Biogenesis |
| CNAG_01091 | -1.116841523 | 5.33E-05 | 0.0005064 | -0.853039461 | 2.18E-23 | 2.13E-22 | RNA Processing & Ribosome Biogenesis |
| CNAG_00111 | -1.10552921 | 6.99E-05 | 0.000641 | -0.09372733 | 0.259600166 | 0.319428091 | RNA Processing & Ribosome Biogenesis |
| CNAG_06684 | -1.100622379 | 0.011078 | 0.0436097 | 1.545216293 | 5.94E-20 | 4.67E-19 | RNA Processing & Ribosome Biogenesis |
| CNAG_07400 | -1.091522137 | 3.13E-06 | 0.0000419 | -0.863126315 | 1.46E-35 | 2.78E-34 | RNA Processing & Ribosome Biogenesis |
| CNAG_00044 | -1.082098085 | 0.000534 | 0.0037023 | -0.602007087 | 1.93E-12 | 8.62E-12 | RNA Processing & Ribosome Biogenesis |
| CNAG_04628 | -1.07439877 | 0.037743 | 0.1131959 | 0.158515008 | 0.050439245 | 0.072380708 | RNA Processing & Ribosome Biogenesis |
| CNAG_00352 | -1.07057086 | 0.069796 | 0.179612 | 0.203980199 | 0.037291545 | 0.054771132 | RNA Processing & Ribosome Biogenesis |
| CNAG_06061 | -1.068908263 | 0.000114 | 0.0009815 | -0.509964429 | 6.74762E-09 | 2.1843E-08 | RNA Processing & Ribosome Biogenesis |
| CNAG_05926 | -1.041191583 | 0.002219 | 0.0122054 | 0.016086063 | 0.854914554 | 0.880984838 | RNA Processing & Ribosome Biogenesis |
| CNAG_01305 | -1.027868283 | 2.84E-05 | 0.0002972 | -0.680262052 | 4.86E-13 | 2.28E-12 | RNA Processing & Ribosome Biogenesis |
| CNAG_07563 | -1.019020608 | 0.029297 | 0.0930775 | -0.894542048 | 2.89E-22 | 2.65E-21 | RNA Processing & Ribosome Biogenesis |
| CNAG_02437 | -1.015749231 | 9.11E-06 | 0.0001084 | 0.557729461 | 7.24089E-07 | 1.94047E-06 | RNA Processing & Ribosome Biogenesis |
| CNAG_00557 | -1.003489329 | 0.069175 | 0.1786176 | 0.02260812 | 0.821646017 | 0.852549846 | RNA Processing & Ribosome Biogenesis |
| CNAG_05455 | -1.002521067 | 7.01E-06 | 0.0000865 | -0.429407143 | 1.47326E-06 | 3.81921E-06 | RNA Processing & Ribosome Biogenesis |
| CNAG_05360 | -1.000742039 | 0.013084 | 0.0502003 | 0.120244017 | 0.242728622 | 0.30098494 | RNA Processing & Ribosome Biogenesis |
| CNAG_01413 | -0.989625088 | 0.003272 | 0.0166914 | -0.31919235 | 7.06508E-05 | 0.000152511 | RNA Processing & Ribosome Biogenesis |
| CNAG_06626 | -0.983794862 | 0.004314 | 0.0206916 | 0.499705225 | 4.01119E-08 | 1.21079E-07 | RNA Processing & Ribosome Biogenesis |
| CNAG_03606 | -0.978903449 | 0.000001 | 0.0000151 | 0.059362269 | 0.450600406 | 0.517733989 | RNA Processing & Ribosome Biogenesis |
| CNAG_01709 | -0.973948882 | 0.004758 | 0.0222215 | -0.464169195 | 1.89798E-09 | 6.48231E-09 | RNA Processing & Ribosome Biogenesis |
| CNAG_03817 | -0.952431567 | 0.003528 | 0.0176136 | 0.683818878 | 3.48714E-08 | 1.05721E-07 | RNA Processing & Ribosome Biogenesis |
| CNAG_00073 | -0.937988756 | 0.001129 | 0.0069727 | -7.51880456 | 4.22E-47 | 4.39E-45 | RNA Processing & Ribosome Biogenesis |
| CNAG_06206 | -0.913386084 | 0.000608 | 0.0041324 | -0.191090796 | 0.086755936 | 0.119285824 | RNA Processing & Ribosome Biogenesis |
| CNAG_00147 | -0.912263706 | 6.25E-05 | 0.000583 | 0.005658109 | 0.955609431 | 0.963724518 | RNA Processing & Ribosome Biogenesis |
| CNAG_00509 | -0.897027778 | 0.000381 | 0.0027553 | -0.370934327 | 1.95231E-06 | 4.99985E-06 | RNA Processing & Ribosome Biogenesis |
| CNAG_05363 | -0.850845649 | 0.005684 | 0.0254731 | -0.263486146 | 0.003418288 | 0.005930667 | RNA Processing & Ribosome Biogenesis |
| CNAG_01734 | -0.841264764 | 0.002297 | 0.0125067 | -0.059371118 | 0.446488243 | 0.513795233 | RNA Processing & Ribosome Biogenesis |
| CNAG_02028 | -0.806312957 | 0.051996 | 0.1445502 | -0.584167143 | 0.000243013 | 0.000490808 | RNA Processing & Ribosome Biogenesis |
| CNAG_00402 | -0.794189459 | 0.00154 | 0.0090342 | -0.728131609 | 3.62E-20 | 2.89E-19 | RNA Processing & Ribosome Biogenesis |
| CNAG_01235 | -0.792141501 | 0.002525 | 0.0134919 | -0.742582902 | 2.15E-17 | 1.41E-16 | RNA Processing & Ribosome Biogenesis |
| CNAG_01833 | -0.7887013 | 0.020018 | 0.0697033 | 0.088972186 | 0.33287364 | 0.397697408 | RNA Processing & Ribosome Biogenesis |
| CNAG_04370 | -0.762656949 | 0.028374 | 0.0909311 | 0.698442095 | 7.55E-12 | 3.21E-11 | RNA Processing & Ribosome Biogenesis |
| CNAG_00527 | -0.756837073 | 0.024626 | 0.0818592 | 0.242230427 | 0.002062567 | 0.003692102 | RNA Processing & Ribosome Biogenesis |
| CNAG_02901 | -0.749213244 | 0.010222 | 0.0407484 | -0.735311586 | 4.86357E-08 | 1.4596E-07 | RNA Processing & Ribosome Biogenesis |
| CNAG_02382 | -0.713277894 | 0.032661 | 0.1010006 | 1.048366981 | 9.84E-21 | 8.12E-20 | RNA Processing & Ribosome Biogenesis |
| CNAG_03965 | -0.712796337 | 0.068001 | 0.1763077 | -0.20286309 | 0.048146944 | 0.06934261 | RNA Processing & Ribosome Biogenesis |
| CNAG_02209 | -0.700302723 | 0.002301 | 0.0125186 | 0.481669804 | 5.11311E-07 | 1.3927E-06 | RNA Processing & Ribosome Biogenesis |
| CNAG_07381 | 0.708868222 | 0.149342 | 0.3077891 | 1.107069831 | 6.94E-15 | 3.75E-14 | RNA Processing & Ribosome Biogenesis |
| CNAG_02162 | 0.751074 | 0.175781 | 0.3464877 | 2.012989192 | 1.45E-60 | 9.26E-59 | RNA Processing & Ribosome Biogenesis |
| CNAG_02564 | 0.752721013 | 0.163838 | 0.3291891 | 0.373577294 | 0.000232734 | 0.000471078 | RNA Processing & Ribosome Biogenesis |
| CNAG_06007 | 0.825749391 | 0.024281 | 0.0811711 | 1.141001109 | 8.35E-15 | 4.48E-14 | RNA Processing & Ribosome Biogenesis |
| CNAG_05627 | 0.851654208 | 0.010047 | 0.0402349 | 0.993060621 | 1.15E-10 | 4.39E-10 | RNA Processing & Ribosome Biogenesis |
| CNAG_03064 | 0.87177425 | 0.024522 | 0.0816677 | 1.449683194 | 1.70E-46 | 5.37E-45 | RNA Processing & Ribosome Biogenesis |
| CNAG_04728 | 0.878691681 | 0.049195 | 0.138367 | 0.559050585 | 4.97E-11 | 1.97E-10 | RNA Processing & Ribosome Biogenesis |
| CNAG_06865 | 1.016132902 | 0.030371 | 0.0960062 | 0.711313758 | 1.12803E-08 | 3.58322E-08 | RNA Processing & Ribosome Biogenesis |
| CNAG_04770 | 1.048654462 | 0.018959 | 0.0668248 | 1.055795027 | 1.66E-33 | 2.88E-32 | RNA Processing & Ribosome Biogenesis |
| CNAG_03625 | 1.138895161 | 0.00036 | 0.0026301 | 0.887275421 | 1.33E-15 | 7.62E-15 | RNA Processing & Ribosome Biogenesis |
| CNAG_01665 | 1.234479989 | 0.011856 | 0.0463042 | 1.413137933 | 3.13E-17 | 2.03E-16 | RNA Processing & Ribosome Biogenesis |
| CNAG_01807 | 1.759244687 | 1.95E-08 | 4.19E-07 | 0.201927882 | 0.021806293 | 0.033267703 | RNA Processing & Ribosome Biogenesis |
| CNAG_07466 | 1.832273287 | 0.002309 | 0.0125421 | 0.833494413 | 4.54081E-09 | 1.49319E-08 | RNA Processing & Ribosome Biogenesis |
| CNAG_03205 | 2.080391637 | 1.61E-13 | 9.72E-12 | 0.779194552 | 6.79585E-08 | 2.00974E-07 | RNA Processing & Ribosome Biogenesis |
| CNAG_01296 | 2.491569698 | 2.14E-12 | 9.55E-11 | 0.930106012 | 4.90E-15 | 2.68E-14 | RNA Processing & Ribosome Biogenesis |
| CNAG_07630 | 1.552295161 | 0.001025 | 0.0064132 | 1.187854317 | 1.16E-45 | 3.49E-44 | RNA Processing & Ribosome Biogenesis |
